# Pilot Study: Evaluating the Impact of Pharmacist Patient-Specific Medication Recommendations for Diabetes Mellitus Therapy to Family Medicine Residents

**DOI:** 10.3390/pharmacy8030158

**Published:** 2020-08-31

**Authors:** Camlyn Masuda, Rachel Randall, Marina Ortiz

**Affiliations:** Daniel K Inouye College of Pharmacy, University of Hawaii at Hilo, Hilo, HI 96720, USA; randall8@hawaii.edu (R.R.); ortiz33@hawaii.edu (M.O.)

**Keywords:** chronic care management, team-based primary care, pharmacist in primary care

## Abstract

Pharmacists have demonstrated effectiveness in managing diabetes mellitus (DM) and lowering hemoglobin A1C (A1C) through direct patient management. Often patients with diabetes and elevated A1C may not be able to come into the clinic for separate appointments with a pharmacist or for diabetes education classes. A novel way that pharmacists can assist in improving the control of patients’ diabetes and improve prescriber understanding and the use of medications for diabetes is by providing medication recommendations to medical residents prior to the patient’s appointment with the medical resident. The results of this pilot study indicate that the recommendations provided to family medicine residents and implemented at the patient’s office visit helped to lower A1C levels, although the population size was too small to show statistical significance. This pilot study’s results support performing a larger study to determine if the pharmacist’s recommendation not only improves patient care by lowering A1C levels but if it also helps improve medical resident’s understanding and use of medications for diabetes.

## 1. Introduction

Pharmacists in ambulatory care clinics have demonstrated effectiveness in improving the health of patients with diabetes mellitus (DM) by lowering hemoglobin A1C (A1C) [1]. A common measure of diabetes control is A1C and the American Diabetes Association (ADA) recommends lowering A1C to help prevent complications [2]. Improving management and the control of a patient’s DM by lowering A1C prevents microvascular and macrovascular complications [3,4]. Sinclair et al. has shown that, in addition to improving patient care, pharmacists also help increase clinic reimbursement from value-based payments for decreasing the number of patients that have uncontrolled DM [5]. Unfortunately, pharmacists are not available in every primary care clinic and are not able to see every patient with uncontrolled DM. In addition, patients may not show up to these appointments because they have challenges getting to the clinic or are fearful to discuss their condition [6,7].

In about one-third of Family Medicine residency programs, pharmacists have been a vital member of the healthcare team and a majority of their time is spent providing pharmacotherapy recommendations [8]. To help improve the management of patients with DM and encourage the appropriate use of newer therapies (e.g., glucagon-like peptide-1 agonists) or complicated medication regimens, such as basal and bolus insulin, providing recommendations to medical residents is another way pharmacists can help improve patient care with long-term benefits. By providing these recommendations prior to a patient’s appointment, the visit is enhanced by decreasing the wait time that is spent while the medical resident consults with the pharmacist and physician preceptor. The medical resident may also benefit from gaining more confidence in the appropriate use of drug therapies for DM. Medical residents come from different medical schools, which do not have consistent training in DM management and when they first start the residency program they have not had the opportunity to chronically manage severely uncontrolled patients with DM in clinic settings [9]. This pilot study was conducted to determine if pharmacist’s recommendations for DM therapy in patients with A1C ≥ 8% given to family medicine residents prior to an upcoming office visit would help improve that patient’s A1C levels and if these recommendations were helpful to the medical resident. 

## 2. Materials and Methods 

This was a prospective pilot, Investigational Review Board waived and privacy board approved quality improvement project conducted at the University of Hawaii at Manoa John A. Burns School of Medicine, Department of Family Medicine and Community Health clinic, located in a rural area in the state of Hawaii. The pharmacist in the clinic was employed by the University of Hawaii at Hilo Daniel K. Inouye College of Pharmacy and had a collaborative agreement and memorandum of understanding to work in the clinic. A postgraduate year one pharmacy resident also assisted with the project. 

A list of patients, 18 years and older, with DM was obtained from the electronic medical record (EPIC) and stored on a secure remote desktop. The electronic medical record automatically generated this report based on diabetes mellitus diagnosis code within the office visit notes, problem list or medical history. The report calculated the percentage of patients with an A1C > 9% because this is a quality metric that is a measure of DM control and the data are reported to organizations, such as the National Committee for Quality Assurance (NCQA) and Medicare as part of the Comprehensive Primary Care Plus Program [10,11]. Although the quality metric included A1C > 9%, this study included established patients with an A1C ≥ 8% to increase the number of patients in the study and include those that had uncontrolled DM. Patients with an A1C < 8% were not selected because certain patients may have a higher target A1C goal of < 8%. ADA guidelines recommend an A1C goal of < 8% in some patients, such as those with advanced complications from diabetes (e.g., albuminuria), chronic conditions or a history of severe hypoglycemia [2]. 

Patients included in the pilot study were established patients, 18 years and older, with Type 2 DM, A1C ≥ 8% and had an upcoming appointment with one of the family medicine residents in the upcoming 6-8 weeks. There were no other additional exclusion criteria. Chart reviews performed on appropriate patients included researching prior DM therapies and patient-specific concerns, such as contraindications, cost, administration concerns, allergies and intolerances. Recommendations for diabetes management were patient-specific, evidence based (ADA or American Association of Clinical Endocrinologist (AACE) diabetes guidelines) [12,13] and cost effective based on patients’ drug coverage. Diabetes management may be multifaceted; therefore, multiple recommendations were given per patient to encompass many possible caveats. If information in the chart did not include medical conditions that may be contraindications for use for medications, the recommendation to the medical resident included verifying with the patient if they had a history of these medical conditions. All recommendations also included medication-specific side effects to monitor. To determine the most cost-effective medications, the pharmacist reviewed the patient’s drug formulary coverage on the internet whenever it was available. In addition to medication recommendations, the pharmacist also included recommendations on ordering pertinent labs (A1C, urine albumin to creatinine ratio, lipid panel, serum creatinine, etc.). The family medicine residents, who were in postgraduate training years 1–3, made the final decision to implement the recommendation based on the information obtained during the visit, which incorporated any contraindications or concerns (e.g., hypoglycemia) presented by the patient. The resident’s final plan for the patient was discussed with their physician preceptor before implementation. To improve consistency of recommendations, only two pharmacists were involved in the study. A post graduate year one pharmacy resident made the initial recommendations and the supervising pharmacist, who is a certified diabetes care and education specialist, reviewed the initial recommendations and made changes to the recommendations when necessary. 

The recommendations were either verbally shared with the family medicine resident, if the resident was in the clinic with the pharmacist 1–3 days before the patient’s office visit, or a message was sent in the electronic medical record. Chart reviews were performed after the office visit and the following data were collected: if recommendations were accepted, if recommendations were not accepted, the reason it was not accepted and the patient’s follow-up A1C drawn closest to the date of the visit. If the office visit note’s assessment and plan section included the recommendation that the pharmacist provided, it was considered implemented. If the recommendation was not documented in the assessment and plan section, it was labelled as a recommendation not implemented. The rest of the office visit note was reviewed to determine reasons for lack of implementation and if no documentation of management of DM was included in the office visit note, the reason for not implementing the recommendation was labelled as not enough time to discuss DM or the patient was seen for another reason/complaint. Recommendations given to the medical residents occurred over a period of 8 weeks from June to August 2019. Changes in A1C percentage for patients that the medical resident implemented the pharmacist’s recommendations (recommendation implemented) were compared to the patients that the medical resident did not implement the recommendations (recommendation not implemented) and analyzed with a paired Student’s t-test. Follow-up A1C results after the office visit and the recommendation given were conducted anywhere from 2 weeks to 8 months later.

The participating family medicine residents were asked to complete a written survey. The following questions were asked: if the recommendations were helpful; if the resident wanted to continue receiving the recommendation; if they preferred receiving recommendations verbally, in writing or both.

## 3. Results

The original report received from the electronic medical record generated 50 unique patients. The pharmacist performed chart reviews on 27 of the 50 patients. Of the 27 patients, six patients had a baseline A1C of < 8% and were excluded from the data analysis. The pharmacist reviewed the chart and made a recommendation for DM therapy for 21 patients; 12 (57%) of the patients were male with an average age of 49 years. Only 3 patients were ≥ 65 years old. See Table 1 for additional baseline characteristics. 

Of the 21 recommendations made, 11 (52%) were sent via electronic message in the medical record system compared to 10 (48%) conveyed to the medical resident verbally. Of those recommendations made, 15 (71%) were implemented by the medical resident. Only 6 (29%) recommendations were not implemented and the reasons for this include that patients that did not show up for their appointment (*n* = 2), patients declined recommendations (*n* = 2) and there was not enough time to discuss DM or the patient was seen for another reason/complaint (*n* = 2).

The A1C collected after the office visit was the one closest to the appointment when the pharmacist recommendation was made. In the recommendation implemented group, the follow up A1C occurred between 0.5–8 months (median 2.75 months) later, and in the recommendation not implemented group, the follow up A1C occurred between 1–3.5 months (median 1.38 months) later. There was a higher number of patients in the recommendation implemented group who had a decrease in A1C (67%) compared to the recommendation not implemented group (50%). The average difference in A1C pre and post study was decreased by 1.3% for the patients that the pharmacist’s recommendation was implemented, and increased by 0.4% in the group in which the recommendation was not implemented (*p* = 0.18). Table 2 includes a comparison of the average A1C levels for all patients and the changes between groups. There were three patients in the recommendation implemented group compared to one patient in the recommendation not implemented group that did not have an A1C done after the office visit. The average A1C calculated was based on the number of patients that had an A1C available after office visit.

Only two family medicine residents out of eleven completed the written survey (18% response rate) and all responses were that the pharmacist’s recommendations were helpful, that they wanted to continue receiving the recommendations and preferred written recommendations compared to verbal.

## 4. Discussion

Of the patients for whom the pharmacist recommendations were implemented, a majority of patients (67%) had a decrease in their A1C, 40% of which had an A1C that was ≤ 8%, which is close to target goal of < 7% for most patients with DM [2]. The average change in A1C from pre and post recommendation decreased by 1.3% in the recommendation implemented group, whereas in the group in which recommendations were not implemented, the average A1C increased slightly (0.4%). These results indicate that pharmacist recommendations given prior to the office visit may help lower A1C levels and improve the control of DM, and are in alignment with improvements in A1C in patients being chronically managed by pharmacists compared to physician care [14]. Ambulatory care pharmacists that implement both direct patient care and provide patient-specific recommendations could help a larger number of patients with DM lower their A1C. 

This study was unique in that it provided patient-specific recommendations based on clinical factors, medication history, and drug formulary coverage. A limitation of this study is that the total population was small in this pilot study, *n* = 21, and the results did not show statistical significance. One of the challenges in getting a higher number of patients in the study is the time it takes to make the patient-specific recommendations and research formulary coverage. Diabetes is a complex disease to manage and many factors, such as medication adherence and lifestyle modifications, need to be assessed before making medication changes/additions. Recommendations were made after extensive chart reviews and included caveats for use, such as assessing patient’s blood glucose levels at the time of the visit, hypoglycemia and assessing if the patient had any contraindications to the medication which may not have been included in the electronic medical record. The pharmacist included many factors regarding the safety and efficacy of medications in the recommendations given and provided alternative plans if other situations occurred when the resident talked with their patient. The family medicine residents assessed the patient at the visit for any other factors that may have not been considered and made the final recommendation based on their findings from that visit. The residents also reviewed and discussed with the physician preceptor, prior to implementing the plan, which helped ensure patient safety. Thus, if there were other factors the family medicine resident obtained during the actual office visit, the recommendations were not implemented. Patients included in the study were identified by a report generated by the electronic medical record system based on the diabetes diagnosis code manually added to the record in the problem list, office visit or medical history. There were some patients with diabetes not included in the report if the physician did not enter a diabetes diagnosis code or if a patient had an incorrect primary care provider assigned to their medical record. However, this would most likely have omitted only a small number of patients. 

Although the lowering of A1C is promising, there were other limitations to this study. Roughly, 19% of patients did not have an A1C drawn after their office visit or scheduled office visit, which may have influenced the average post-recommendation A1C levels. These patients did not complete lab tests or did not come in for follow up, despite recommendations and reminder calls to schedule follow up appointments. This rate is similar to average no show appointment rates of 24% reported for family practice clinics, which may indicate this is similar to real life practice [6]. 

ADA guidelines recommend checking an A1C every 3 months if a patient’s A1C is not controlled [2]. However, in practice it is difficult to get all patients to have their A1C drawn exactly 3 months after the visit. For the purposes of this quality improvement pilot study, the A1C drawn closest to the date of the office visit where the recommendation was made ensured a consistent data point. The median of 2.75 months after the office visit for recommendations implemented is close to the recommendation to check at 3 months. However, the recommendation not implemented group’s median time A1C value after the visit was 1.38 months, which may not be an appropriate comparison to the group in which the recommendation was implemented, since it was done so soon after the visit. Further analysis comparing the A1C 3 months after the recommendation versus the one done closest to the date of the office visit would be a helpful subgroup analysis to determine if the recommendations that are accepted do lower A1C, as it should be lower for both data points. 

Another factor affecting post-recommendation A1C levels is adherence. Even though the family medicine resident implemented the pharmacist recommendation and prescribed new medications or altered the medication regimens, the patient may not have been compliant. Other factors, such as improper administration technique for injectable medications, although not accounted for, are worth mentioning.

This study did not create a formal protocol or treatment algorithm for the recommendations, which may cause differences in recommendations. To limit the differences, all recommendations were based on the ADA and/or AACE treatment algorithms, and a pharmacy resident provided the initial recommendation that was reviewed by one pharmacist. A protocol/treatment algorithm should be included in the larger study to ensure the consistency of recommendations as more pharmacists and pharmacy residents will be involved. 

Other challenges conducting this study were duplicate pharmacist efforts made when patients rescheduled their appointment with another family medicine resident. The reasons the patient rescheduled with another resident included patient preference or that the original resident was not available (scheduled in another rotation/clinic, on vacation or sick). In these situations, the pharmacist sent the recommendation to the new family medicine resident assigned to see the patient. In some cases, the pharmacist was not aware of the change in provider until after the visit, due to last minute changes in the schedule, and the new provider did not receive the recommendation. Determining a way to link the recommendation to the patient to have it available to different family medicine residents will help to ensure the different providers have access to the recommendation.

Another objective of the study was to assess if the recommendations made by the pharmacist were helpful to the family medicine residents. Although feedback was positive and residents requested to continue receiving the recommendations, having only two surveys completed was a limitation for this part of the study. Higher survey response rates could have been achieved by distributing the survey electronically or by sending reminders the day of or the day after the visit. Supplementary studies should include survey questions that assess if the recommendations improve the residents’ understanding and implementation of DM medications. Improving family medicine residents’ understanding and implementation of DM medications could translate to far-reaching, long-term benefits, as this could improve therapy inertia and will further improve patient care.

Based on the promising results from this pilot, additional studies with a larger population are warranted. Additions to a future trial besides those listed above, that would improve the validity of the results, would be to add a control group, defined as those patients with DM and an A1C ≥ 8% that were seen by the medical residents in the same time frame but did not receive the pharmacist’s recommendations. 

## 5. Conclusions

The results of this pilot study are promising and indicate further studies are warranted to confirm that patient-specific pharmacist’s recommendations for DM management given either verbally or written, to a family medicine resident prior to a patient’s appointment is an effective method to reduce patient’s A1C. 

## Figures and Tables

**Table 1:** Baseline Characteristics.

	All Patients Chart Reviewed (*n* = 21)	Pharmacist Recommendation Implemented (*n* = 15)	Pharmacist Recommendation Not Implemented (*n* = 6)
Age			
Between 18 and 65 years	18 (86%)	13 (87%)	5 (83%)
≥65 years	3 (14%)	2 (13%)	1 (17%)
Gender			
Female	9 (43%)	7 (47%)	2 (33%)
Male	12	8	4
Average body weight (kg)	93.4	88.6	105.3
Average baseline number of medications for diabetes	2.14	2.33	2.08
Number of patients on insulin	12	8	4

**Table 2:** Average A1C levels and change of levels.

	All Patients Chart Reviewed (*n* = 21)	Pharmacist Recommendation Implemented (*n* = 15) *	Pharmacist Recommendation Not Implemented (*n* = 6)
Effect on A1C			
A1C decreased	13 (62%)	10 (67%)	3 (50%)
A1C increased	4 (19%)	2 (13%)	2 (33%)
No A1C available	4 (19%)	3 (20%)	1 (17%)
A1C ≤ 8 post recommendation	7 (33%)	6 (40%)	1 (17%)
Average A1C prior to recommendation	10.6%	10.4%	11%
Average A1C post recommendation **	9.7%	9.1%	11.4%
Difference in A1C	−0.9%	−1.3% (*p* = 0.18)	+0.4%

* One A1C result was > 14%, which was rounded to 14% for calculating the average for the recommendation implemented group. ** Average calculated based on number of patients that had an A1C available after office visit.
